# Efficacy and safety of visually guided laser balloon versus cryoballoon ablation for paroxysmal atrial fibrillation: a systematic review and meta-analysis

**DOI:** 10.3389/fcvm.2023.1229223

**Published:** 2023-08-22

**Authors:** Wenyi Ye, Qian Chen, Guangci Fan, Xinbin Zhou, Xiao Wang, Wei Mao, JuanJuan Li

**Affiliations:** ^1^ The First Affiliated Hospital of Zhejiang Chinese Medical University (Zhejiang Provincial Hospital of Chinese Medicine), Hangzhou, China; ^2^ Qingdao Hiser Hospital Affiliated of Qingdao University (Qingdao Traditional Chinese Medicine Hospital), Qingdao, China; ^3^ Department of Cardiology, Zhejiang Hospital, Hangzhou, China

**Keywords:** paroxysmal atrial fibrillation, catheter ablation, laser balloon ablation, cryoballoon ablation, meta-analysis

## Abstract

**Background:**

Newly developed catheter ablation (CA) techniques, such as laser balloon ablation (LBA) and cryoballoon ablation (CBA), have been introduced in recent years and emerged as valuable alternatives to conventional radiofrequency CA strategies for paroxysmal atrial fibrillation (PAF) patients. However, evidence comparing LBA and CBA remain controversial. Thus, we conducted this meta-analysis to assess the efficacy and safety between these two techniques.

**Methods:**

Scientific databases (PubMed, Embase) and relevant websites (the Cochrane Library, ClinicalTrials.gov) were systematically searched from inception to March 2023. The primary outcomes of interest were the AF recurrence and the procedure-related complications. Secondary outcomes included procedural time, fluoroscopy time, and left atrial (LA) dwell time.

**Results:**

Seven clinical trials with a total of 637 patients were finally enrolled. No significant differences were found between LBA and CBA in terms of AF recurrence [16.3% vs. 22.7%, odds ratio (OR) = 0.66, 95% confidence interval (CI): 0.42–1.05, *p* = 0.078] or total procedural-related complications (8.4% vs. 6.4%, OR = 1.33, 95% CI: 0.71–2.51, *p* = 0.371). LBA had a significantly longer procedural time [weighted mean difference (WMD) = 38.03 min, 95% CI: 13.48–62.58 min, *p* = 0.002] and LA dwell time (WMD = 46.67 min, 95% CI: 14.63–78.72 min, *p* = 0.004) than CBA, but tended to have shorter fluoroscopy time.

**Conclusions:**

LBA and CBA treatment have comparable efficacy and safety for PAF patients. LBA was associated with longer procedural and LA dwell times compared with CBA. Further large-scale studies are warranted to compare these two techniques with the newest generations.

**Systematic Review Registration**: https://www.crd.york.ac.uk/prospero/display_record.php?RecordID=426513, identifier (CRD42023426513).

## 1. Introduction

Atrial fibrillation (AF) is a common cardiac arrhythmia, and patients with AF are known to be at increased risk of morbidity and mortality (1). Catheter ablation (CA) has been the most effective therapeutic approach in restoring and maintaining sinus rhythm for symptomatic AF patients, and pulmonary vein isolation (PVI) has been recognized as the cornerstone and fundamental therapeutic strategy of CA (2).

Several balloon-based catheter ablation techniques, including laser balloon ablation (LBA) and cryoballoon ablation (CBA), have been introduced in recent years and emerged as valuable alternatives to conventional radiofrequency CA strategies (3). Previous studies have demonstrated that balloon-based CA techniques not only have comparable efficacy and safety outcomes but also provide several superiorities, such as shorter procedural and fluoroscopy durations, especially for paroxysmal AF (PAF) patients (4, 5).

Several studies have compared the characteristics, efficacy, and safety between LBA and CBA as initial therapies for PAF patients; however, the results remain controversial (6, 7). Therefore, the aim of the present meta-analysis was to investigate the efficacy, safety, and procedural characteristics between LBA and CBA for PAF patients in light of the latest evidence.

## 2. Materials and methods

### 2.1. Search strategy and selection criteria

Scientific databases (PubMed, Embase) and relevant websites (the Cochrane Library, ClinicalTrials.gov) were systematically searched from inception to March 2023. The following keywords and the corresponding variants were used: “laser balloon,” “cryoballoon,” and “paroxysmal atrial fibrillation.” In addition, the reference lists of all eligible articles were manually checked for potentially relevant studies. Full-text articles in English that directly compared LBA and CBA in the treatment of PAF and reporting interested outcomes were included.

### 2.2. Data collection and quality assessment

Data extraction and quality assessment were performed by two authors (WY and GF) independently with divergences resolved with a third author (JL). The following data were extracted: author's name, publication year, sample size, participant characteristics, ablation protocol, duration of follow-up, and outcomes of interest. Two authors working independently assessed the risk of bias using the Cochrane Collaboration tool (8) for randomized controlled trials (RCTs) and the Risk of Bias in Non-randomized Studies of Interventions (ROBINS-I) tool (9) for the non-randomized studies. The Cochrane Collaboration tool included six domains: random sequence generation, allocation concealment, blinding for outcome assessment, incomplete outcome data, selective reporting, and other bias. And the ROBINS-I tool included seven domains: confounding, selection of participants into the study, classification of interventions, deviations from intended interventions, missing data, measurement of outcomes, and selection of the reported result.

### 2.3. Primary and secondary outcomes

The primary outcomes of interest were the AF recurrence, defining as AF/atrial flutter/atrial tachycardia documented on the ECG or Holter continuing longer than 30 s during follow-up, and the procedure-related complications. Secondary outcomes included procedural time, fluoroscopy time and left atrial (LA) dwell time.

### 2.4. Statistical analysis

Statistical analysis was performed using the STATA software package (version 14.1 for macOS; STATA Corporation, College Station, TX, USA). Categorical variables were described as *n* (%) and continuous variables were described as median and standard deviation (SD). Odds ratio (OR) and weighted mean difference (WMD) with the 95% confidence interval (CI) were calculated to demonstrate the summary statistics for comparisons between LBA and CBA. The random-effects model was applied. The between-study heterogeneity was assessed using the inconsistency index (*I*^2^) statistic (*I*^2^ < 25% = low, *I*^2^: 25%–50% = moderate, and *I*^2^ > 50% high heterogeneity). When significant heterogeneity was present, possible causes were investigated. The likelihood of publication bias was analyzed by funnel plots graphically and by Egger's and Begg's tests statistically. The protocol for this systematic review and meta-analysis was registered on PROSPERO (doi: 10.15124/CRD42023426513).

## 3. Results

### 3.1. Eligible studies and characteristics

**Figure 1:**
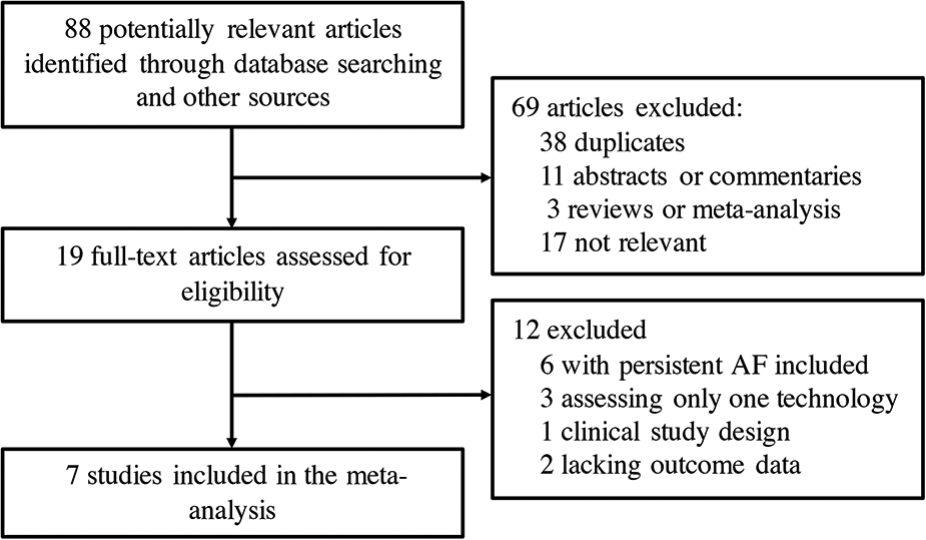
Flow chart of the systematic literature research.

**Figure 2:**
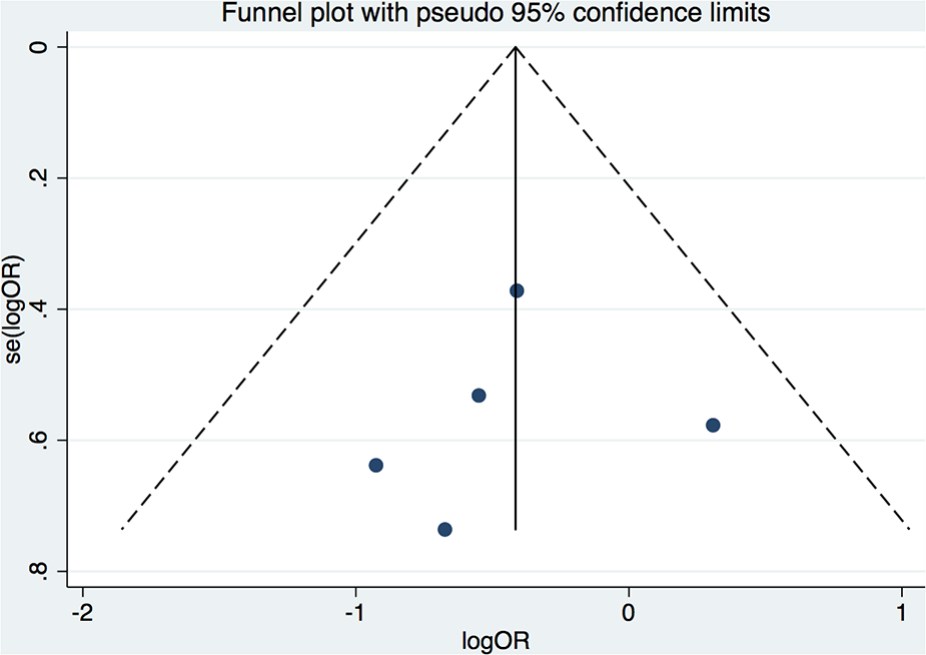
Funnel plot for the studies included.

Seven clinical trials (6, 7, 10–14) from 88 potentially relevant studies were finally included in the meta-analysis (Figure 1). A total of 637 patients receiving initial ablation for PAF (LBA, *n* = 311 vs. CBA, *n* = 326) were studied. The main characteristics of the studies and the participants included are reported in Table 1. Briefly, across the trials, two studies (10, 12) were RCTs while the rest studies were non-randomized prospective clinical trials. There were 311 patients in the LBA group and 326 patients in the CBA group. LBA with the first-generation laser balloon (LB1) was performed in all studies. CBA with the first-generation CB (CB1) was applied in one study (6), while CBA with the second-generation CB (CB2) were applied in three studies (11, 13, 14). The mean age of the patients ranged from 57.6 to 73 years. The mean left ventricular ejection fraction (LVEF) ranged from 61.8% to 70% and the mean left atrium dimeter (LAd) ranged from 36 to 43 mm. Median follow-up length was 12.4 months. All the included studies had good qualities according to the Cochrane Collaboration tool (8) and ROBINS-I tool (9) (Supplementary Tables S1, S2). No significant publication bias was found by funnel plot or Egger's and Begg's tests based on the primary outcomes (Egger's: *p* = 0.789; Begg's: *p* = 0.462) (Figure 2).

**Table 1:** Baseline characteristics of the included studies.

Study	Year	Study type	N	Mean age (years)	Male (%)	Mean LVEF (%)	Mean LAd (mm)	Hypertension (%)	DM (%)	LBA protocol	CBA protocol	Monitoring protocol	Mean follow-up
Ohkura et al. (7)	2022	Prospective	65	67.2	75.4	NR	37.8	47.7	16.9	LB1	NR	NR	6 months
Kobori et al. (11)	2021	Prospective	100	67	69	62.5	36	47	NR	LB1	CB2	Visits (ECG, 24 h Holter monitoring) at 1, 3, 6, 9, and 12 months	12 months
Yano et al. (14)	2021	Prospective	111	73	57.7	70	42.5	58.6	14.4	LB1	CB2	Visits (ECG, 24 h Holter monitoring and portable ECG) every 2 weeks	350 days
Tsyganov et al. (13)	2015	Prospective	100	62.5	63	NR	43	64	16	LB1	CB2	Visits (ECG, 24 h Holter monitoring) at 3, 6, and 12 months	12 months
Casella et al. (10)	2014	RCT	55	57.6	72.7	61.8	41.8	40	NR	LB1	CB1/2	Visits (ECG, 24 h/7 days Holter monitoring) at 1, 3, 6, and 12 months	12 months
Schmidt et al. (12)	2013	RCT	66	65.5	NR	60	40	75.8	6.1	LB1	NR	NR	21 months
Bordignon et al. (6)	2013	Prospective	140	63	66	63	39.9	62	12	LB1	CB1	Visits (72 h Holter monitoring) at 3, 6, 9, and 12 months	12 months

DM, diabetes mellitus; NR, not reported.

### 3.2. Primary end points

**Figure 3:**
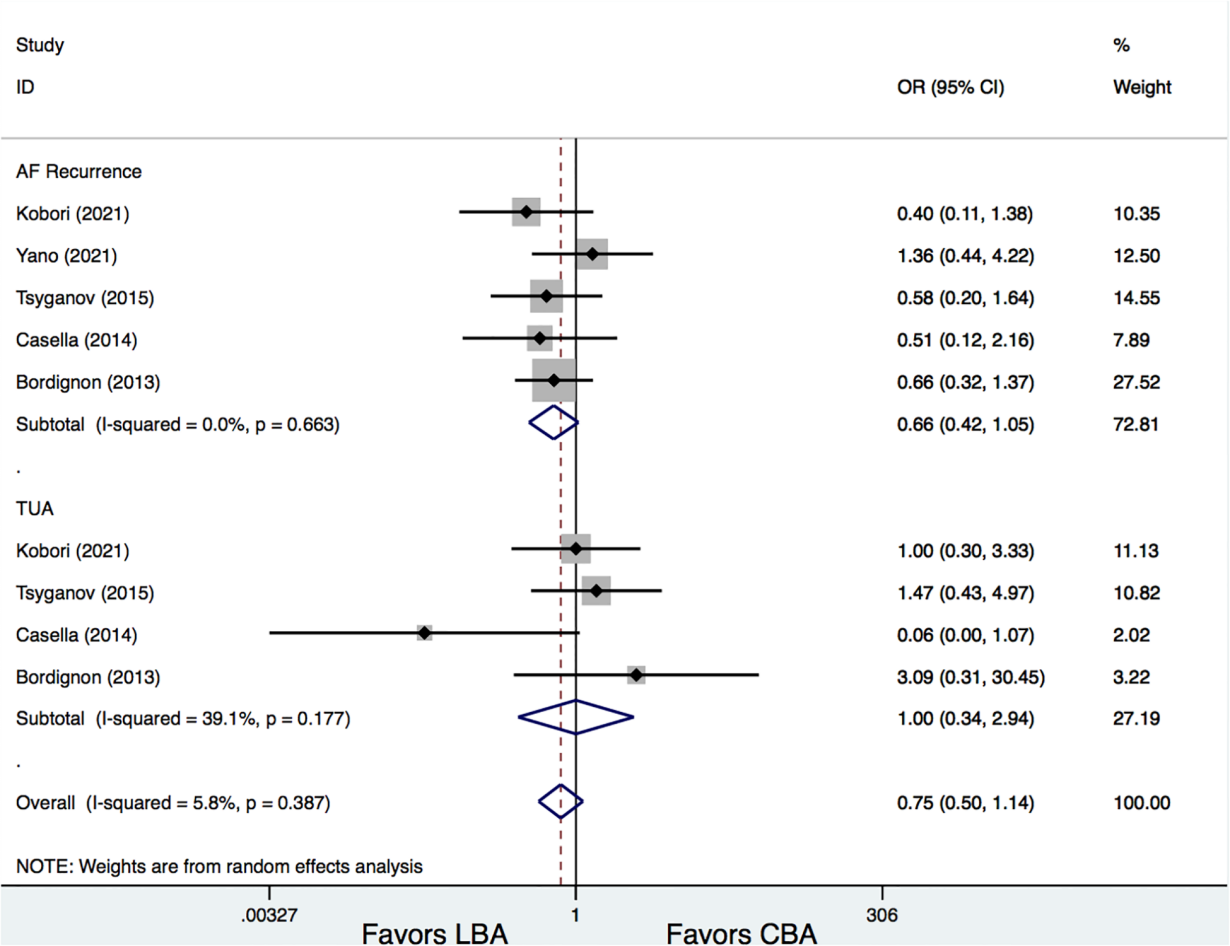
Forest plots for the outcome of AF recurrence and TUA.

Of the included trials, five studies (6, 10, 11, 13, 14) provided information on AF recurrence after LBA and CBA treatments. Results demonstrated that there was no significant difference between LBA and CBA regarding AF recurrence (16.3% vs. 22.7%, OR = 0.66, 95% CI: 0.42–1.05, *p* = 0.078). No significant heterogeneity was detected (*I*^2 ^= 0%) (Figure 3).

Four studies (6, 10, 11, 13) additionally provided data regarding the needs of touch-up ablation (TUA) during LBA and CBA procedures and showed that LBA and CBA had comparable TUA rates (8.4% vs. 10.7%, OR = 1.00, 95% CI: 0.34–2.94, *p* = 1.00). No significant heterogeneity was detected (*I*^2 ^= 39.1%) (Figure 3) Additional meta-regression analyses did not show significant associations between AF recurrence and the study and patient characteristics, such as year of publication, ablation protocols, participant number, mean LAd, mean LVEF, monitoring protocols, and follow-up lengths (*p* > 0.05 for all), whereas the leave-one-out analysis was further performed and showed that, when the study by Yano et al. (14) was removed, LBA treatment was associated with significantly lower AF recurrence rate compared with that of CBA (OR = 0.57, 95% CI: 0.35–0.95, *p* = 0.03).

**Figure 4:**
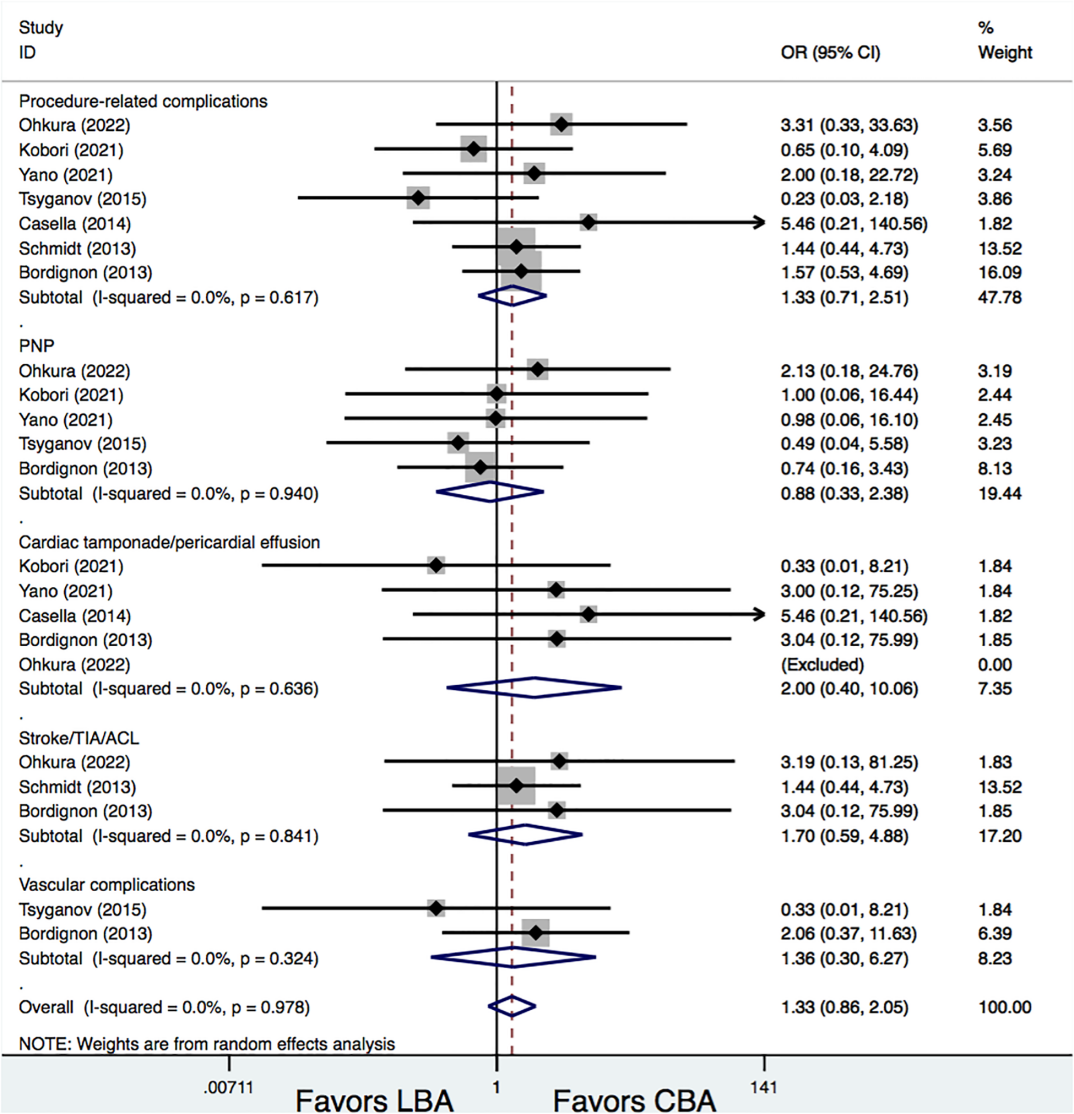
Forest plots for the outcome of procedural-related complications.

All the studies included provided information on procedure-related complications. Results demonstrated that the total procedure-related complications rates were similar between the LBA and CBA treatments (8.4% vs. 6.4%, OR = 1.33, 95% CI: 0.71–2.51, *p* = 0.371). Additional subgroup analyses were conducted according to different complication types, and the results showed that, there were no significances between LBA and CBA regarding phrenic nerve palsy (PNP) (2.6% vs. 2.8%, OR = 0.88, 95% CI: 0.33–2.38, *p* = 0.807), cardiac tamponade/pericardial effusion (1.0% vs. 0.3%, OR = 2.00, 95% CI: 0.40–10.06, *p* = 0.399), stroke/transient ischemic attacks (TIA)/asymptomatic cerebral lesions (ACL) (3.2% vs. 1.8%, OR = 1.70, 95% CI: 0.59–4.88, *p* = 0.325), and vascular complications (1.3% vs. 0.9%, OR = 1.36, 95% CI: 0.30–6.27, *p* = 0.689). No significant heterogeneities were detected for all the comparisons (*I*^2^ = 0%) (Figure 4).

### 3.3. Secondary end points

**Figure 5:**
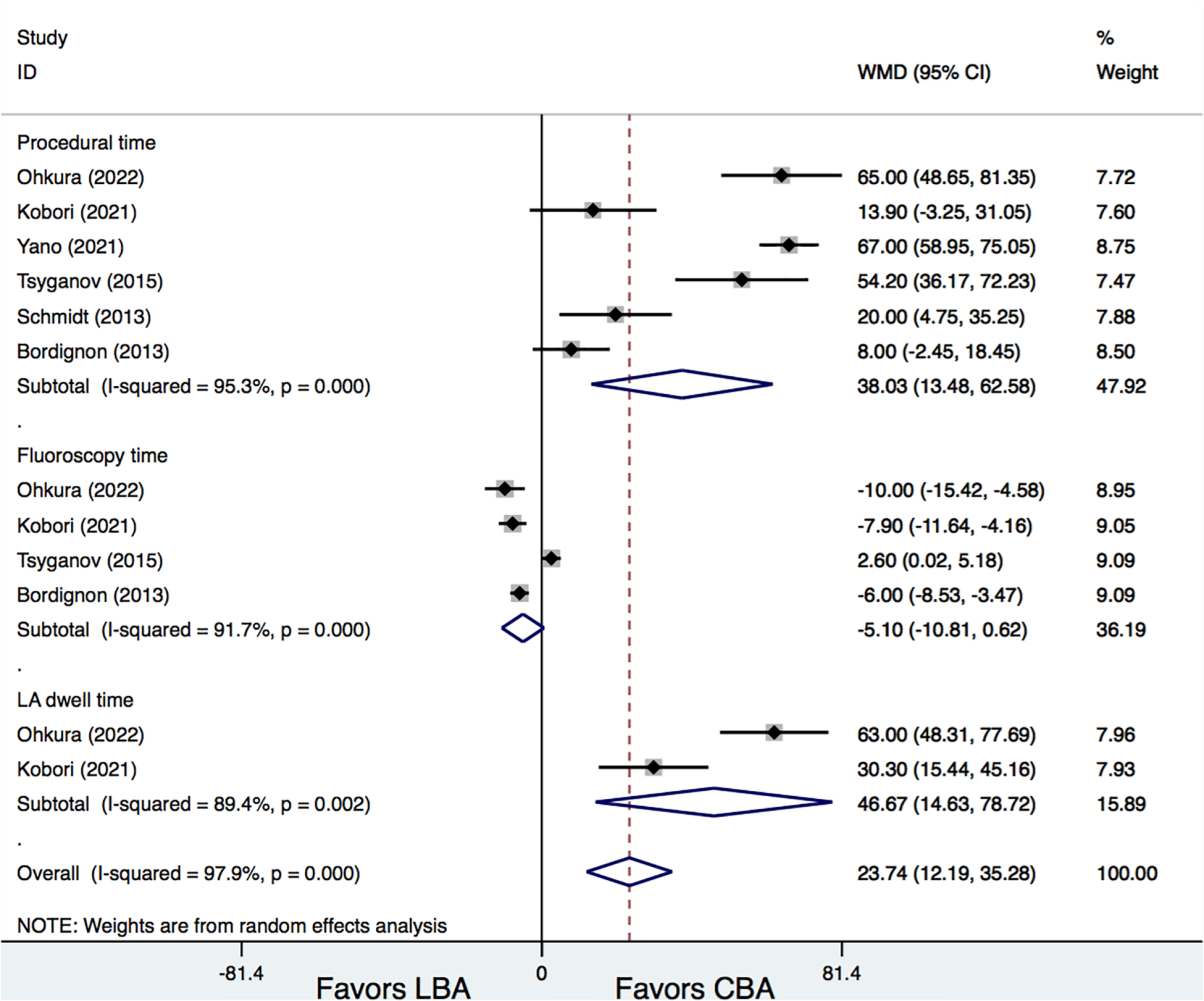
Forest plots for the secondary outcomes.

Six studies (6, 7, 11–14) provided information on procedural time of LBA and CBA. Results demonstrated that, LBA had a significantly longer procedural time than CBA (WMD = 38.03 min, 95% CI: 13.48–62.58 min, *p* = 0.002). In addition, LBA also needed a longer LA dwell time than CBA (WMD = 46.67 min, 95% CI: 14.63–78.72 min, *p* = 0.004) (Figure 5). No significant difference was found regarding fluoroscopy time between LBA and CBA therapy (WMD = −5.10 min, 95% CI: −10.81 to 0.62 min, *p* = 0.081). However, significant heterogeneities were detected for comparisons (*I*^2 ^= 95.3%, 89.4%, and 91.7%, respectively). Meta-regression analysis was further conducted, whereas no significantly associations between procedural time and the study and patient characteristics were detected (*p* > 0.05 for all). Additional subgroup and leave-one-out analysis were also performed and showed that, when the study by Tsyganov et al. (13) was removed, LBA treatment was associated with significantly shorter fluoroscopy time compared with that of CBA (WMD = −7.04 min, 95% CI: −9.00 to −5.08 min, *p* = 0.00) (Figure 5).

## 4. Discussion

This meta-analysis included seven studies with a total of 637 patients. The major findings were as follows: (1) LBA had a non-significant lower AF recurrence rate compared with CBA (16.3% vs. 22.7%); (2) The needs of TUA during procedure were comparable between the two technologies; (3) LBA and CBA showed comparable safety profile; (4) LBA had significantly longer procedural and LA dwell time than CBA, but tends to have shorter fluoroscopy time.

Current guidelines recommend PVI by means of CA as treatment for drug-refractory PAF (4). Point-by-point radiofrequency catheter ablation (RFCA) has been a standard of care for PVI; however, it still has shortcomings, such as technical complexity, long procedure time, high rates of complications, and long learning curve (15). The balloon-based CA technologies including LBA and CBA have been introduced in recent years to overcome the complexity of the conventional point-by-point RFCA procedure, which also have shown simplicity, reproducibility, and similar effectiveness compared with RFCA, especially for PAF patients (4, 16). For persistent AF patients, RFCA has shown advantages, when additional ablations of atrial myocardium beyond pure PVI are needed, though the STAR-AF2 trial proved that pure PVI was non-inferior to more extensive atrial ablation in patients with persistent AF (17). However, persistent AF has a more complex pathophysiologic basis than PAF, and pure PVI is sufficient in most cases for PAF patients. Thus, the balloon-based CA techniques have advantages over RFCA for PAF patients, with comparable efficacy but shorter procedure duration.

In the present study, LBA and CBA were directly compared for PAF patients and demonstrated similar AF recurrence at a median of 12.4 months. However, it should be noted that AF recurrence seemed to be lower after LBA compared with CBA during further sensitivity analysis. Possible reasons may be that, first, though low heterogeneity was detected (*I*^2 ^= 0%) for this outcome, the definitions of AF recurrence and rhythm monitoring strategies were non-uniform across the studies. The rates of freedom from AF recurrences in the present study were 83.7% in the LBA group and 77.3% in the CBA group, which were higher than that in the studies using continuous rhythm monitoring strategies by Rovaris et al. (18) and Andrade et al. (19). They reported that the 1-year freedom from recurrences was 66.9%, 81.0%, and 86.8% considering any, 5.5-h, and 24-h cut-off duration after LBA (18), and was 52.2% and 51.7% after 4-min CBA and 2-min CBA, respectively (19). Continuous rhythm monitoring is thought to be essential and the most accurate approach to assess the post-procedure AF burden and the true value of certain AF ablation techniques (20). However, all the included trials in this study used only Holter ECGs as monitoring tools. This intermittent monitoring tool is thought to be a very limited technique to truly assess AF recurrences, which may inevitably cause bias (18). Second, except the study by Yano et al. (14), non-significant lower AF recurrences were seen after LBA in the remaining studies. It was reported that the PVI lesions created by LBA had a durability of 86% at 3 months during repeat mapping (21). In addition, in patients with clinical recurrence and repeat procedures, the chronic isolation rates were reported to be only 32% after CBA, compared with that of 59% after LBA (6). The relatively high durability of electrical PVI may indicate better arrhythmic outcomes of LBA compared with CBA.

Though the lesion size created by CBA was reported to be larger than that by LBA, it was not seen to be correlated with clinical outcome after a single procedure during 1-year follow-up (22). In a multicenter, randomized trial of LBA that compared with RFCA, 94.1% of patients were able to achieve electrical PVI with the LBA alone (16), whereas in the STOP AF Trial, only 83% PVI was achieved with the CBA alone, and additional ablation was required (23). However, in the present study, data were limited to compare the acute PVI rates, but the incidence of additional TUA was found to be comparable between these two techniques, which were consistent with the previously work by Seki et al., comparing hot balloon, LBA, and CBA for both PAF and persistent AF patients (24).

As for the safety profile, LBA and CBA had comparable overall complication rates, with no significant differences in complication types. The total procedure-related complication rate in the present study (7.4%) was a little higher than that reported in the study by Chun et al. (5.4%), which investigated the complications in 3,000 AF patients after CBA and LBA procedures (25). However, the total complications rates of both LBA and CBA in the present study were still lower than that reported in their initial randomized comparisons to standard RFCA (11.8% for LBA in the HeartLight Study and 12.8% for CBA in the FIRE and ICE trial) (4, 16). Possible reasons may be the different definitions of procedural complications across these trials, as ACL was recognized as a complication in the present study, while dyspnea, gastrointestinal complication, and contrast media reaction were defined as complications in other trials (4, 16).

PNP was a common periprocedural complication of balloon-based procedures for AF (26). Though it was reported that PNP recovery time was significantly longer during LBA than that during CBA, due to their greatly different lesion formations (27), the majority of PNP were usually transient, most of which could recover during follow-up.

Different from PNP, cardiac tamponade and pericardial effusion are serious perioperative complications. It was reported that, the incidence of cardiac tamponade was approximately 1.3% in all the CA techniques for AF (28). The incidence of cardiac tamponade for LBA and CBA procedure was reported to be 0.1% in the study with 3,000 AF patients in a high-volume center (25). The incidences of cardiac tamponade and pericardial effusion in the present study were inconsistent with the published data. However, it should be noted that, the majority of events of cardiac tamponade/pericardial effusion occurred in the LBA group (3/4) in the present study. In a large RCT trial that compared LBA and RFCA, the incidence of cardiac tamponade/pericardial effusion was reported to be 1.2% (16), whereas in the FIRE and ICE trial, this incidence was only 0.3% for CBA (4).

These results indicated that LBA may be more vulnerable to cardiac tamponade/pericardial effusion than CBA. Possible reasons may be that the CBA procedure was navigated through a guidewire but LBA was not; in addition, unlike the single-shot CBA technique, more catheter manipulations were required for LBA in the LA with segment-by-segment ablation (27). Operator experiences and the generations of the techniques used may also be important potential reasons. As it was reported, adverse event endpoints were improved with increased operator experience over 15 LBA cases, demonstrating the learning curve effect with the newly introduced technologies such as LBA (16, 29).

These new balloon-based CA techniques were designed to reduce the complexity of the conventional RFCA procedure, which could greatly reduce the total procedural time (20). In the present study, LBA was shown to have a significantly longer procedural time and LA dwell time than CBA. As mentioned above, the non-compliant CBA system represents as a single-shot PVI tool, in contrast to the segment-by-segment ablation procedure by LBA (27). LBA could offer direct PV visualization and provide a more precise regional energy titration, at the cost of more procedural times. In addition, LBA lacks the technique for real-time PV potentials recording as CBA, contributing to increased procedural time when PVI validation was needed, especially in the case of failed first-round PVI (27).

On the other hand, owing to the inherent visually guiding characteristic of LBA during procedure, less fluoroscopy time was supposed to be required (6). In our study, LBA was found to be associated with a non-significantly shorter fluoroscopy time compared with CBA, whereas during further sensitivity analysis, LBA showed a significant reduction in fluoroscopy time. This is reasonable as CBA requires a serial angiogram to achieve optimal PV occlusion, while LBA offers visually guided balloon control and deployment. It should still be noted that the first-generation laser balloon was applied in all the studies included. It was reported that the more compliant second- and third-generation laser balloon has been applied, offering an automated continuous lesion deployment, which contributes to the improvements in PV occlusion, energy delivery, and shorter procedural time (30, 31). Thus, future larger prospective multicenter randomized studies are warranted to reveal the efficacy, safety profiles, and procedure characteristics between LBA and CBA, especially for these techniques with the newest generations.

The current study has a number of limitations. First, the number of trials and the total sample size of the present analysis were relatively small, and large-scale clinical trials, especially RCTs, comparing LBA and CBA, were rare. Second, AF recurrence definitions and monitoring strategies across trials were non-uniform, and all the included studies used Holter ECG monitoring rather than continuous rhythm monitoring, which is limited in assessing AF recurrences and may cause possible bias. However, we performed an additional meta-regression analysis to further investigate the potential moderator effect of the study and patient features on AF recurrence. Third, LB1 was applied in all the studies and CB2 was exclusively used in only three studies. As LBA with the second- and third-generation and CBA with the third-generation have been introduced and widely applied in recent years, and the improvement of the LBA between the first and the second/third generation was enormous, the results need to be further updated to provide up-to-date evidence. Finally, considerable heterogeneities were detected in the analysis of the secondary outcomes; though meta-regression and sensitive analyses have been performed, the interpretation should still be taken with caution.

## 5. Conclusions

LBA and CBA treatments have comparable efficacy and safety in terms of AF recurrence and procedure-related complications as initial therapies for PAF patients. CBA therapy is associated with a shorter procedural time and LA dwell time compared with that of LBA, while LBA therapy tends to have shorter fluoroscopy time. Further large-scale RCTs with the newest generations of LBA and CBA are needed to provide up-to-date evidence.

## Data Availability

The raw data supporting the conclusions of this article will be made available by the authors, without undue reservation.
