# Supplementary material for: Efficacy and safety of visually guided laser balloon versus cryoballoon ablation for paroxysmal atrial fibrillation: a systematic review and meta-analysis
Source: Front Cardiovasc Med. 2023 Aug 22;10:1229223. doi: 10.3389/fcvm.2023.1229223 (PMC10478246; doi:10.3389/fcvm.2023.1229223)
Supplement: Supplementary file 1 [file Table1.docx]

**SupplementaryTable1 Risk of Bias with Cochrane Collaboration tool**

| Study | Inadequate allocation sequence generation? | Inadequate allocation concealment? | Inadequate blinding? | Incomplete outcome data? | Selective outcome reporting? | Risk of other bias? |
| --- | --- | --- | --- | --- | --- | --- |
| Casella[10] | No | No | Yes | No | No | No |
| Schmidt[12] | No | No | Yes | No | No | No |

**SupplementaryTable2 Risk of Bias with Non-randomized Studies of Interventions (ROBINS-I) tool.**

|  | Confounding | Selection of participants into the study | Classification of interventions | Deviations from intended interventions | Missing data | Measurement of outcomes | Selection of the reported result | Overall risk of bias |
| --- | --- | --- | --- | --- | --- | --- | --- | --- |
| Ohkura[7] | Low | Low | Moderate | Low | Moderate | Low | Low | Moderate |
| Kobori[11] | Low | Low | Low | Low | Low | Low | Low | Low |
| Yano[14] | Low | Moderate | Low | Low | Moderate | Low | Low | Moderate |
| Tsyganov[13] | Low | Moderate | Low | Low | Moderate | Low | Low | Moderate |
| Bordignon[6] | Low | Low | Low | Low | Low | Low | Low | Low |
